# Chronic and Intermittent Hyperglycemia Modulates Expression of Key Molecules of PI3K/AKT Pathway in Differentiating Human Visceral Adipocytes

**DOI:** 10.3390/ijms22147712

**Published:** 2021-07-19

**Authors:** Ewa Świderska, Justyna Strycharz, Adam Wróblewski, Piotr Czarny, Janusz Szemraj, Józef Drzewoski, Agnieszka Śliwińska

**Affiliations:** 1 Department of Medical Biochemistry, Medical University of Lodz, 92-215 Lodz, Poland; justyna.strycharz@umed.lodz.pl (J.S.); adam.wroblewski@stud.umed.lodz.pl (A.W.); piotr.czarny@umed.lodz.pl (P.C.); janusz.szemraj@umed.lodz.pl (J.S.); 2 Central Hospital of Medical University, 92-213 Lodz, Poland; jozef.drzewoski@umed.lodz.pl; 3 Department of Nucleic Acids Biochemistry, Medical University of Lodz, 92-213 Lodz, Poland; agnieszka.sliwinska@umed.lodz.pl

**Keywords:** adipocytes, adipogenesis, IRS1, PI3K, AKT2, GLUT4, PTEN, miR, hyperglycemia, diabetes

## Abstract

Background: Due to its prominence in the regulation of metabolism and inflammation, adipose tissue is a major target to investigate alterations in insulin action. This hormone activates PI3K/AKT pathway which is essential for glucose homeostasis, cell differentiation, and proliferation in insulin-sensitive tissues, like adipose tissue. The aim of this work was to evaluate the impact of chronic and intermittent high glucose on the expression of biomolecules of insulin signaling pathway during the differentiation and maturation of human visceral preadipocytes. Methods: Human visceral preadipocytes (HPA-V) cells were treated with high glucose (30 mM)during the proliferation and/or differentiation and/or maturation stage. The level of mRNA (by Real-Time PCR) and protein (by Elisa tests) expression of IRS1, PI3K, PTEN, AKT2, and GLUT4 was examined after each culture stage. Furthermore, we investigated whether miR-29a-3p, miR-143-3p, miR-152-3p, miR-186-5p, miR-370-3p, and miR-374b-5p may affect the expression of biomolecules of the insulin signaling pathway. Results: Both chronic and intermittent hyperglycemia affects insulin signaling in visceral pre/adipocytes by upregulation of analyzed PI3K/AKT pathway molecules. Both mRNA and protein expression level is more dependent on stage-specific events than the length of the period of high glucose exposure. What is more, miRs expression changes seem to be involved in PI3K/AKT expression regulation in response to hyperglycemic stimulation.

## 1. Introduction

It is believed that visceral adipose tissue (VAT) may play a key role in the development of T2DM [1,2]. VAT surrounds the abdominal viscera in the mesentery and omentum. It produces and releases a large number of adipocytokines that influence both VAT and other tissues [2,3]. It is suggested that hyperglycemia (HG), a typical marker of diabetes causing nutrient stress, may alter the production of adipocytokines and change biochemical pathways including insulin signaling [3,4]. It was demonstrated that during chronic HG (CHG), adipocytes (Ads) are not capable of storing energy excess and the process of new Ads formation, adipogenesis (ADG), is interrupted [5]. As a result, hypertrophy of Ads occurs making them dysfunctional and exhibiting insulin resistance phenotype. Furthermore, it is suggested that similarly to CHG, intermittent HG (IHG) is also involved in Ads formation abnormality and, as a result, in the progression to T2DM. Recent data indicate a prominent role of epigenetics in this process [6,7].

In insulin-sensitive cells (Ads, myocytes, hepatocytes) insulin signaling has been implicated in several cellular processes such as proliferation, differentiation, and metabolism [8]. In Ads, the insulin-dependent PI3K/AKT pathway is a positive regulator of terminal differentiation and a crucial player in metabolic response to high-glucose levels. Attachment of the insulin to its receptor (insulin receptor-INSR) activates the pathway. This is followed by the insulin receptor substrate (IRS) phosphorylation. There are 6 isoforms of IRS, however, in Ads, IRS1 is suggested to play a major role [9]. IRS binds to the kinase regulatory subunit of PI3K (PI3K-R), the central molecule of the pathway. This, in turn, causes activation of the catalytic PI3K (PI3K-C) subunit that converts phosphatidylinositol (4,5)-biphosphate (PIP2) to phosphatidylinositol (3,4,5)-triphosphate (PIP3) [8]. The main insulin pathway regulator, PTEN, (phosphatase and tensin homolog), inhibits further signal transduction by PIP3 dephosphorylation [10]. PIP3 recruits AKT kinase and its activators: PDK1 (phosphoinositide-dependent kinase-1) and mTORC2 (mammalian target of rapamycin 2) to the cell membrane [8,11]. Active AKT is one of the key downstream molecules of insulin signaling controlling many cellular processes. Among the isoforms of AKT, AKT2 is expressed predominantly in insulin-sensitive tissues [12]. In Ads, AKT2 controls glucose uptake by phosphorylation of AS160 substrate protein. This in turn promotes GLUT4 translocation to the cell membrane. Cell surface externalization of GLUT4 allows glucose to be transported through this protein into Ads. Furthermore, AKT is established as an essential player in Ads differentiation in mouse embryonic fibroblasts and 3T3-L1 preadipocytes (pAds) [13]. AKT has been shown to regulate ADG via interplaying with the mTOR signaling pathway which promotes lineage commitment, clonal expansion, and terminal differentiation of pAds to mature Ads [14]. What is more, AKT2 has been shown to regulate ADG also by targeting FOXO1 (forkhead box-O1) and inhibiting its transcriptional activation. FOXO1 regulates the expression of peroxisome proliferator-activated receptor gamma (*PPARγ*) and CCAAT enhancer-binding proteins (*C/EBPs*), two critical transcriptional regulators of ADG [15].

Along with recent studies suggesting that Ads may possess the memory of exposure to environmental factors, Ads may preserve the one of exposure to HG (CHG and IHG) [5,7,16]. This allowed us to hypothesize, that as a result of high-glucose stimulus Ads may exhibit not fully reversible changes in the expression of biomolecules involved in insulin signaling. What is more, since HG has been recognized as a factor strongly influencing epigenetic regulatory mechanisms (DNA methylation, histones methylation, and acetylation, miRs, lnc-RNAs) in VAT, we speculated that one of the possible mechanisms underlying these changes may occur via miRs’ expression modulations. MiRs constitute a class of short non-coding RNAs that evoked translational repression by mRNA degradation and as a result, impact the level of specific proteins. They exhibit the unique ability to have multiple targets and thus, one miR can regulate a large number of protein-coding genes (Table 1). Furthermore, miRs expression changes are suggested to be involved in the pathophysiology of various diseases, including metabolic disorders [17,18,19].

This in vitro study aimed to evaluate whether CHG and IHG affect biomolecules such as IRS1, PI3K, AKT2, PTEN, and GLUT4 in visceral p/Ads during the ADG at both the mRNA and protein level. Moreover, we determined the expression of miR-29a-3p, miR143-3p, miR-145-5p, miR-152-3p, miR-186-5p, miR-370-3p, and miR-374b-5p, indicated as epigenetic regulators of the expression of the tested biomolecules of insulin signaling (see Table 1).

## 2. Results

### 2.1. Evaluation of the Gene Expression Profile of Insulin Signaling Pathway Molecules under CHG and IHG

**Figure 1:**
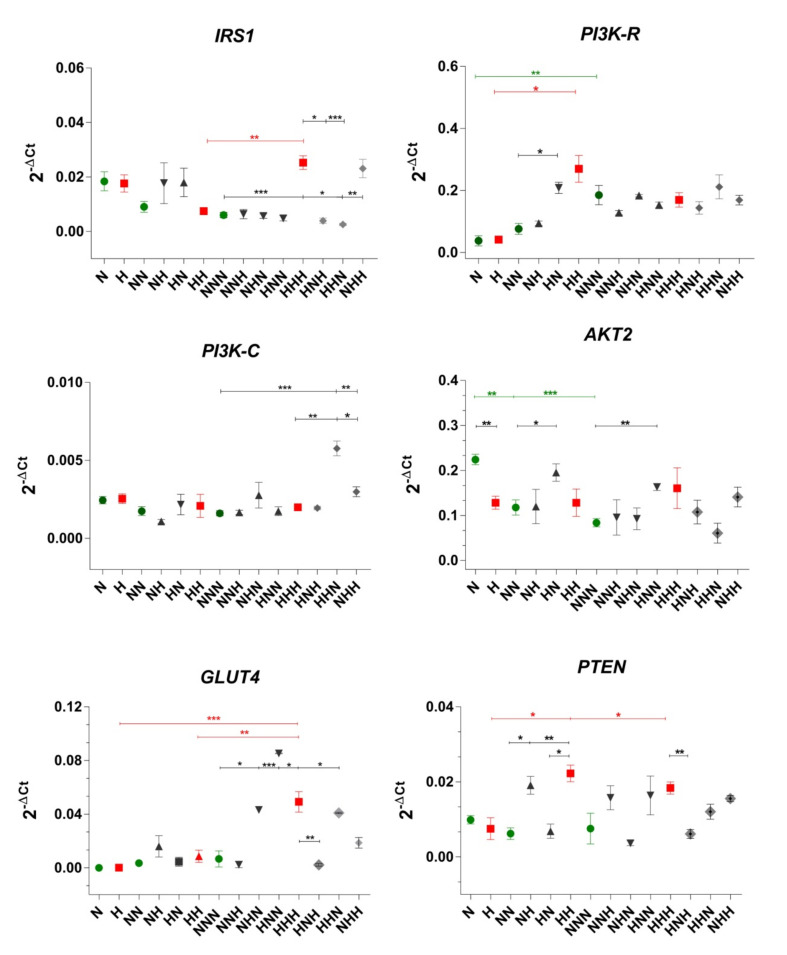
mRNA expression levels of *IRS1, PI3KR, PI3KC, PTEN, AKT2*, and *GLUT4* evaluated after completion of proliferation, differentiation, and maturation of visceral cellscultured in chronic/intermittent normoglycemic (N) and hyperglycemic (H) conditions. Data are expressed as mean ± SEM in green (NG), red (chronic HG), dark grey (single HG hit), and light grey (double HG hit). Differences in expression levels between two particular culture variants were evaluated using a two-tailed *t*-test. One-way ANOVA with a post-hoc Tukey test was used for the calculation of statistical significance of changes observed during ADG in NG and HG. (***): *p* ≤ 0.001; (**): *p* ≤ 0.01; (*): *p* ≤ 0.05.

Analyzing changes of mRNA expression that occurred in the insulin signaling pathway during in vitro ADG in chronic normoglycemic (NG) conditions (Figure 1), we noted expression alterations in the *PI3K-R* and *AKT2*. In the case of *AKT2* expression decreased at subsequent stages of ADG (most apparent after the maturation stage), while the opposite trend was observed for *PI3K-R* (significant increase after maturation). This may imply both of these molecules to be the signaling node that is crucial for ADG [38]. ADG in CHG evoked mRNA changes in *PTEN* and *GLUT4* (Figure 1). Namely, their expression was stable during proliferation and differentiation and increased after achieving the mature stage. Comparing ADG in CHG to NG, the expression of two vast molecules *IRS1*, and *GLUT4* was significantly elevated in mature Ads (NNN vs. HHH).

To conclude, during the process of ADG in NG and HG we observed that mRNA expression mostly changed after the maturation stage. None of the molecules differed in expression between pAds and differentiated cells considering both NG and CHG. Taking all into account, these observations may suggest that effects of high glucose do not manifest until cells reach maturity. This type of response of Ads to HG may suggest the occurrence of the phenomenon of the so-called metabolic memory. IHG also evoked alterations in mRNA expression of analyzed molecules (Figure 1). The stimulus introduced individually (single HG hit) in differentiated Ads caused alterations only in *PTEN* expression in the HN variant (rise in comparison to NN, downregulation in comparison to HH). When HG was introduced at the differentiation stage (NH) we did not observe any expression changes of the studied genes.

Considering single HG hit in mature Ads, HG had the weakest impact when introduced during the maturation phase. In this scenario, the expression profile was similar to the NNN variant. Interestingly, both HNN and NHN variants showed significant upregulation of *GLUT4* comparing to the NNN probe. Furthermore, HG hit introduced during the differentiation stage resulted in a *PI3K-R* increase in mature Ads.

Variants treated with double HG hit were compared both to probes treated with NG and CHG. In comparison to NNN cells, we revealed increased *PI3K-C* expression when HG was maintained during proliferation and differentiation (HHN). Furthermore, we also noted a pronounced increase of *IRS1* and *PI3K-C* expression in NHH-treated cells.

While comparing double HG hit-treated cells to HHH variant, mRNA level drop was found for *IRS1, PTEN, GLUT4*, and mRNA level rise for *PI3K-C*. In the case of the HNH variant, we observed a decline in *IRS1* and *GLUT4* expression. The HHN probe displayed a similar expression profile as was observed for NHH one. For both variants, we noted the increased expression of *PI3K-C* and decreased expression of *PTEN*.

To summarize, IHG had an impact on each analyzed molecule, except for *AKT2* which maintained a stable level. Moreover, even after glucose level normalization, the expression profile of insulin pathway biomolecules remained changed, especially in mature Ads. This may suggest that the effects of HG may be delayed (changes are not visible in differentiated Ads, but are marked in mature cells).

### 2.2. Evaluation of Insulin Signaling Proteins Expression during ADG in CHG and IHG

**Figure 2:**
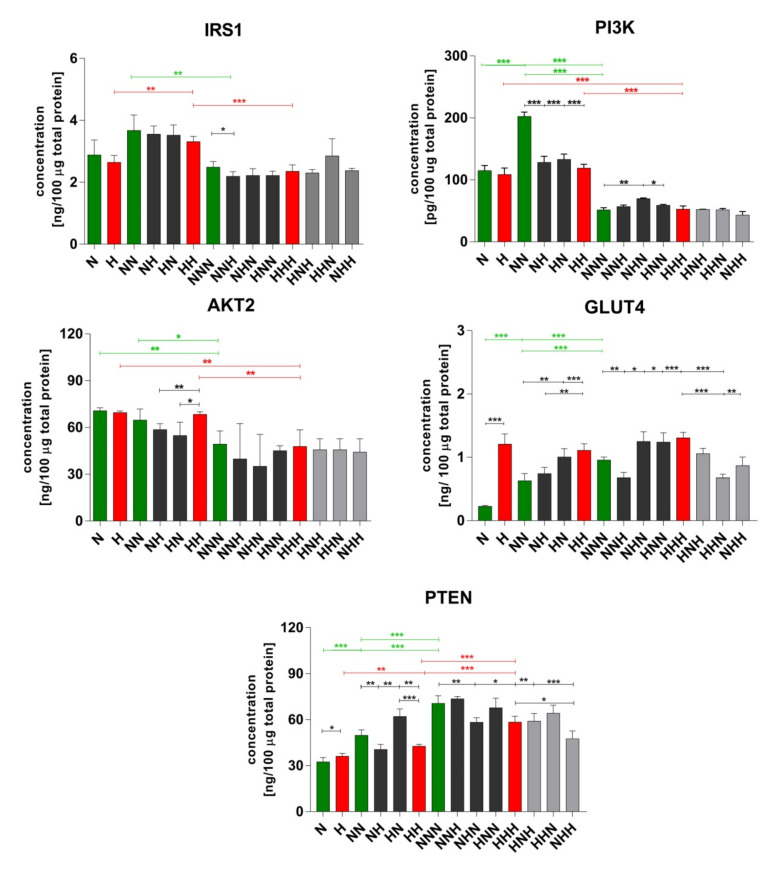
Protein expression profiles of IRS1, PI3K, AKT2, GLUT4, and PTEN were evaluated after completion of proliferation, differentiation, and maturation of visceral cells cultured in chronic/intermittent normoglycemic (N) and hyperglycemic (H) conditions. Data are expressed as mean ± SEM in green (NG), red (chronic HG), dark grey (single HG hit), and light grey (double HG hit). Differences in expression levels between two particular culture variants were evaluated using a two-tailed *t*-test. One-way ANOVA with a post-hoc Tukey test was used for the calculation of statistical significance of changes observed during ADG in NG and HG. (***): *p* ≤ 0.001; (**): *p* ≤ 0.01; (*): *p* ≤ 0.05.

Figure 2 shows changes in insulin pathway proteins’ expression during ADG in NG and CHG. We observed that during ADG in NG PI3K and IRS1 revealed the highest expression after completion of differentiation stage, then it dropped significantly after maturation. The expression of AKT2 decreased significantly after the maturation stage. PTEN and GLUT4 expression were picking up after each phase, reaching the highest level after cell maturation.

Considering ADG in CHG, GLUT4 expression was maintained at a stable level during the whole process. IRS1 exhibited the highest protein level after Ads differentiation. For both PI3K and AKT2 protein level was stable during 1st and 2nd stage, and after maturation, their expression significantly decreased. PTEN exhibited a similar trend during ADG to the one in NG conditions, being shown as a gradual expression increase after each ADG stage.

Analyzing differences between ADG in CHG and NG, we observed PI3K, GLUT4, and PTEN protein expression alterations. In the case of PI3K, significant down-expression was observed after the differentiation stage (HH vs. NN). It seems that more susceptible to the influence of increased glucose concentration was PTEN protein expression, where changes were visible both after the proliferation stage (rise in comparison to N) and differentiation (drop in comparison to NN). CHG-treated cells had a significantly higher level of GLUT4 protein at each stage than cells grown in NG. As GLUT4 is considered as a marker of ADG, this may suggest that HG accelerated differentiation.

Alterations in insulin signaling proteins expression caused by IHG are shown in Figure 2 (black and grey bars). The analysis of a single HG stimulus on differentiated Ads (HN, NH variants) revealed that expression fluctuations in almost all analyzed molecules (except for IRS1). PI3K protein level distinctly dropped in both HN and NH variants in comparison to NN. For AKT2, there were no changes in protein level in comparison to the NN, however, a significant reduction was observed in HN and NH in relation to HH. Taking into account the changes occurring within the GLUT4, we noted a similarity between HN and HH variants as well as NH and NN variants. This supports the hypothesis that the effects of HG may be postponed. PTEN protein expression decreased in NH variant (vs. NN) and increased in HN one (vs. both NN and HH).

Next, we analyzed variants of mature Ads treated with a single HG stimulus (HNN, NHN, NNH) in comparison to NNN. We found that, when HG was introduced at the maturation stage (NNH), the expression of IRS1 and GLUT4 was dropped. On the other hand, HNN and NHN variants showed over-expression of PI3K and GLUT4 protein. What is more, the NHN variant additionally exhibited PTEN down-regulation.

Afterward, we considered how the doubled stimulation of visceral cells with HG differed from NG milieu. These conditions resulted in PTEN protein (HNH and NHH) and GLUT4 protein expression drop (HHN).

Lastly, we compared double HG-hitted mature Ads (HNH, HHN, NHH) with variants treated with CHG (HHH). In this part of our experiment, significant changes were noted for PTEN and GLUT4 protein expression. Analyzing PTEN, we observed an expression decrease in NHH. Considering GLUT4, HHN, and NHH variants showed down-regulated protein expression. Interestingly, the HNH variant exhibited the same protein expression profile for each of the analyzed molecules as cells treated with CHG (HHH).

In conclusion, the most significant changes caused by the IHG occurred when a single hit of HG was introduced (at any stage). Double HG-hit resulted in alterations only in downstream molecules (GLUT4 and PTEN). Furthermore, the IRS1 and the AKT2 seemed to be the least sensitive to IHG stimulus.

### 2.3. Expression of Selected miRs That Target Insulin Signaling Pathway Molecules during ADG in CHG and IHG

The apparent differences between expression at the mRNA and protein levels indicate the existence of other mechanisms involved in the expression regulation of biomolecules of the insulin pathway. Therefore, we decided to check whether miRs that target mRNAs of *IRS*, *AKT*, *PI3KR*, *PTEN*, *GLUT4* (see Table 1) are under influence of CHG and IHG.

**Figure 3:**
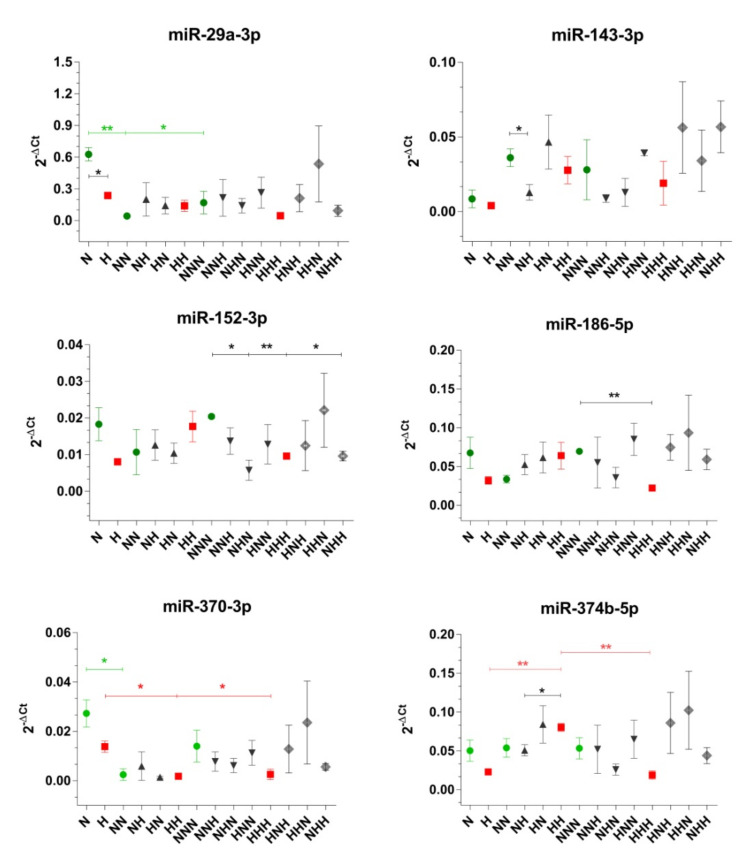
Expression profiles of miR-29a-3p, miR-143-3p, miR-152-3p, miR-186-5p, miR-370-3p and miR-374b-5p evaluated after completion of proliferation, differentiation, and maturation of visceral cells cultured in chronic/intermittent normoglycemic (N) and hyperglycemic (H) conditions. Data are expressed as mean ± SEM in green (NG), red (chronic HG), dark grey (single HG hit), and light grey (double HG hit). Differences in expression levels between two particular culture variants were evaluated using a two-tailed *t*-test. One-way ANOVA with a post-hoc Tukey test was used for the calculation of statistical significance of changes observed during ADG in NG and HG. (**): *p* ≤ 0.01; (*): *p* ≤ 0.05.

Among the molecules tested, during ADG in NG conditions significant expression changes were observed only for miR-29a-3p and miR-370-3p (Figure 3). Expression of these molecules was the highest after the proliferation stage, then dropped, and remained unchanged in differentiated and mature Ads. As shown in Table 1, miR-29a-3p targets each of the analyzed genes of insulin signaling. Its expression profile during NG may partially explain divergences between mRNA and protein levels of PI3K, GLUT4, PTEN. For these molecules, we observed an increase in protein expression between the 1st and 2nd stages, however, this trend was not observed at the mRNA level. Furthermore, miR-29a-3p targets *AKT2* and its expression changes during ADG may explain why the protein level of AKT2 was stable between NN vs. N, while at mRNA level, we observed significant differences. Although miR-370-3p is suggested to target *IRS1*, its expression changes during ADG did not reflect mRNA-protein discordances in our experimental design.

The expression profile of miR-370-3p under CHG exhibited a similar pattern as during ADG in NG. It also corresponded to observed IRS1 protein down-regulation in pAds in comparison to differentiated Ads, which was not seen at the mRNA level. Furthermore, CHG resulted in higher expression of miR-374b-5p after the completion of differentiation in comparison to expression level after other stages. This may be connected with PI3K protein drop in mature Ads (not observed at mRNA level) and weakened PTEN protein expression in differentiated Ads in comparison to mRNA level.

Comparing ADG in NG and CHG, we observed that HG resulted in the down-expression of miR-29a-3p after the proliferation step. This expression change may partially correspond to both AKT2 and GLUT4 inconsistencies in mRNA-protein correlation at this point of ADG (H vs. N). Moreover, CHG evoked an expression drop of miR-152-3p and miR-186-5p in mature Ads (in comparison to NNN). Although miR-152-3p is suggested to target *IRS* and *PTEN*, in our experiment, these miR changes do not seem to be connected with their expression. On the other hand, the observed change in miR-186-5p level may to some extent be linked with PTEN expression.

In Figure 3, we present how IHG affected analyzed miRs expression. Not all considered molecules responded to periodic changes of glycemic conditions (no changes in the case of miR-29a-3p, miR-186-5p, and miR-370-3p). However, in differentiated Ads, when HG was introduced during differentiation (NH), we observed down-regulation of expression of miR-143-3p (comparing to variants treated with NG) and miR-374b-5p (in comparison to HH). Although both of these miRs target insulin signaling genes (Table 1), their expression changes during ADG in IHG did not support their post-transcriptional control of these genes. What is more, in mature Ads, we noted a decrease of miR-152-3p in NHN and NHH variants vs. NNN, which may to some extent be a reason for IRS1 differences in mRNA and protein expression.

To sum up, the obtained data suggest that the studied miRs at least partially regulate the analyzed insulin pathway molecules. MiR-29a-3p seems to be a crucial one for the physiological process of ADG by regulating the expression of almost all analyzed molecules. What is more, miR-29a-3p is a well-known T2DM-related miR. In our research, miR-29a-3p may to some extent be responsible for expression alignment between cells cultured in NG and CHG, thus suggesting its key role as an insulin signaling regulator. Furthermore, only expression changes of miR-374b-5p, miR-186-5, and miR-152-3p can be, to some extent, related to predicted earlier gene expression changes in Ads in response to HG. Finally, although not all observed in our study changes in miR expression may be connected with expression changes of analyzed insulin signaling genes, they still seem to be involved in the response of Ads to HG.

## 3. Discussion

The main goal of our study was to explore whether and how CHG and IHG affect the expression of chosen molecules involved in the PI3K/AKT pathway on each step of visceral ADG. As a point of reference, we assumed changes occurred during ADG in NG.

### 3.1. ADG in CHG

The association between HG and its connection to the insulin-signaling pathway dysfunction in multiple tissues is well described [2,4,10,39,40]. High glucose concentrations may alter insulin signaling in Ads, myocytes, hepatocytes, and endothelial cells (ECs). Subsequently, these changes may lead to reduced AKT activity resulting in the inhibition of GLUT4-mediated glucose influx [41,42,43]. Although glucose uptake in these tissues occurs also through GLUT-1, which provides constitutive insulin-independent glucose uptake, attenuation of downstream insulin signaling at PI3K/AKT in these tissues has been implicated in the development of insulin resistance and T2DM. What is more, PI3K/AKT also plays a crucial role in regulating many physiological cellular processes, and its impairment results in, among others, deterioration of cell differentiation [13,44,45]. To our best knowledge, it is the first study that explored how HG affects this pathway in human visceral Ads during the terminal ADG process.

We previously [5] demonstrated that the high-glucose level impairs the ADG, significantly accelerating the process and resulting in morphological changes in Ads formation [5]. It is therefore not surprising that analyzed conditions also caused changes in insulin signaling, which, apart from the metabolic response, is closely related to Ads differentiation. In earlier studies on AT and skeletal muscle, the expression of *PI3K/AKT* molecules under the influence of HG was downregulated [46,47]. In muscles in response to HG *IRS1* was found to be down-regulated along with distraction of insulin signaling [47]. In isolated rat’s Ads, CHG in the presence of insulin diminishes glucose transport and this effect appears to be associated with a post–insulin receptor dysfunction, but without detectable changes in total expression of GLUT4 [48,49]. Intriguing is, therefore, that our research demonstrates that CHG resulted in particular mRNA (*IRS1*, *PI3K-R*, *GLUT4*) overexpression in comparison to ADG under physiological conditions. It may result in enhanced glucose uptake and lipid droplet accumulation, and as a consequence, increased cell size which was noted in our previous study [5]. The only decrease in mRNA was observed in *AKT2* expression after proliferation, but this effect was not observed at the protein level. This ties well with earlier findings where total AKT protein was not significantly altered in HUVEC cultured in HG compared with 5 mM D-glucose [41]. In the paper cited above, also total PI3K expression was not changed by HG, but in our experiment, we observed PI3K drop in differentiated Ads. The disparity in obtained results may be explained by several differences in experimental design and tissue specificity. Especially it is worth mentioning that in HUVECs, PI3K/AKT signaling plays a crucial role in survival, proliferation, microvascular permeability, and angiogenesis but not in glucose uptake. In HUVECs, *GLUT4* is not expressed, and glucose transport is carried out through GLUT1 which is insulin-independent [41,50].

The results of our study indicate down-regulation of pathway inhibitor, PTEN, in response to CHG at all stages, with additional significant up-regulation of GLUT4 production. Obtained during our study data prevents us from concluding that in our research we are dealing with a weakened insulin signal in newly formed Ads in CHG conditions. Based on our research, we may imply that Ads can adapt to long-term HG stimulus by enhancing insulin signaling. A similar observation has been noted by Laybutt et al. [51]. They concluded that chronic glucose infusion results in enhanced AT glucose uptake, lipogenesis, and insulin action in rats [51]. Moreover, our data suggest that HG alone (without additional insulin stimulation) may not be enough to evoke a cellular insulin resistance state in Ads. This ties well with the fact that AT is a main player during metabolic adaptive processes, and (in contrary to other insulin-sensitive tissues, like muscle) can rapidly remodel in response to environmental inputs [51,52]. On the other hand, it is possible that the pathway impairment mechanism does not involve expression inhibition of insulin-related genes, but only their activity or cellular localization, as was observed in HUVECs [41]. What is more, knowing that HG accelerates ADG and lipid droplets formation (already visible at the stage of proliferation, what was shown in the previous study), high expression of GLUT4 at each stage confirms our conclusion that GLUT4 may be an indicator of Ads maturity [5]. Furthermore, our results support the hypothesis that HG enhances the ADG process at least partially through an increase in the expression of the PI3K/AKT-dependent pathway [53].

Finally, among analyzed miRs, miR-370-3p and miR-374b-5p expression changed during ADG in CHG. MiR-370-3p is suggested to be an important predictor of metabolic syndrome presence [17]. The dysregulation of miR-374b-5p has been implicated in several disorders, including obesity, calcific aortic stenosis, and ischemic stroke [54]. Their expression ties well with observed protein-mRNA changes of their target genes (*IRS1*, *PI3K*, *PTEN*) in our research. What is more, miR-29a-3p may play an important role in blocking the effect of HG on Ads. MiR-29a-3p is a well-known T2DM-related miR and it has been suggested to interact with all analyzed insulin signaling molecules (Table 1). Its expression increases in AT and Ads upon diabetes and HG. In earlier studies, exposure of 3T3-L1 Ads to increasing glucose concentrations (up to 25 mM) resulted in an overall overexpression of miR-29a when compared with control (5 mM) [18]. Our results reveal a contrary trend (however only in pAds). This effect points out variation in molecular response to stimulus between different cell lines. Overall, our findings are in accordance with accumulating evidence suggesting the possible regulatory role ofmiRs in multiple processes involved in obesity, including pAds ADG and insulin sensitivity [19]. What is more, our results imply that miR-29a-3p, miR-370-3p, and miR-374b-5p may be involved in insulin sensitivity and insulin signaling.

### 3.2. ADG in IHG

The previous research on HG effects mostly focused only on the effect of CHG. In fact, there is also another important phenomenon of the repeated fluctuation in blood glucose.IHG may be followed by pathological changes which are even more deleterious than ones caused by CHG [55,56,57]. This effect is usually connected with the fact that exposure to IHG induces a metabolic memory [58,59]. Obtained results are, for our best knowledge, the first to show the role of an IHG on PI3K/AKT pathway molecules in visceral Ads. This data may give a preliminary image of metabolic memory of pathological changes which occurred as a result of HG.

Similar to the effect of CHG, periodic stimulus resulted mostly in mRNA expression upregulation. These results stay contrary to the earlier findings of Meugnier et al. They have demonstrated that IHG induces a global downregulation of gene expression in subcutaneous AT, including insulin signaling molecules like *AKT2* [46]. However, observed differences may be explained by differences in the analyzed material. The increased expression seems to be a characteristic response of visceral Ads for HG stimulus. What is more, expression alterations were seen not only after the particular stage during which HG was introduced, but even after glucose normalization. Further, the effect seems to be postponed in time (alterations are significant mostly one stage after introducing HG). Overall, our data are in accordance with findings reported by Andersen et al. [16] and partially support our earlier findings [5,7,60] that visceral Ads exhibit signs of metabolic memory. Obtained results suggest that miR-143-3p, miR-374-5p, and miR-152-3p may take part in preserving toxic effects of HG in Ads, however, the expression only of miR-152-3p may be related to genes analyzed in our study. Collectively, these results demonstrate that mentioned miRs are involved in insulin sensitivity and insulin signaling regulation while being directly or indirectly modulated in vitro by IHG.

Furthermore, considering protein expression data of insulin signaling molecules, it should be pointed out that PI3K and AKT2, the crucial molecules for differentiation, were strongly affected by IHG. Moreover, IHG has a more substantial impact on AKT2 expression than CHG, thus supporting the hypothesis of metabolic memory in Ads. These results partially tie with studies on heart tissue, which show that fluctuating blood glucose has a more significant impact on the decreased level of p-AKT expression than stable HG level. On the other hand, the aforementioned study showed that total AKT was stable under both CHG and IHG [61].

Finally, it is worth mentioning, that IHG not always affected Ads. NNH and HNH showed no significant changes in comparison to NNN (mRNA), suggesting that HG has a crucial impact when introduced during the differentiation stage. Taken together, our results, both at the mRNA and protein levels, imply that the expression level of analyzed molecules is more dependent on stage-specific events than the length of the period of HG exposure.

### 3.3. Study Limitations

There are few major limitations of this study that could be addressed in future research. First, it should be noted that our study provides only a preliminary in vitro effect of CHG and IHG on the PI3K/AKT pathway in visceral Ads. In order to perform an in-depth analysis, the functionality of the tested molecules should still be checked.

Secondly, we need to clarify that obtained data cannot be considered as an analysis of insulin signal transduction. As most of the molecules in insulin signaling are kinases, the next step should be to study the phosphorylated forms of the protein. Moreover, despite the demonstrated increased expression of GLUT4, transport of glucose into the Ads occurs only when GLUT4 is present at the cell membrane, so its localization in the cell should be investigated as well. We also need to add that beyond miRs, there are several different epigenetic (for example, lncRNAs, which also affect miRs or RNA methylation) mechanisms which may be a potential reason for mRNA-protein expression divergences. Also, numerous post-transcriptional and translational mechanisms may influence the efficiency of translation.

Lastly, cells used in this research were obtained from donor of unknown post- and prenatal history of exposure to metabolic stressors. Moreover, we did not start differentiation protocol from mesenchymal stem cells, whose pool is preserved much longer than this of committed pAds, which make them particularly sensitive to various stimuli throughout life. Therefore, we believe, that to obtain further support for the existence of metabolic memory in Ads, our study design should be implemented on adipose-derived mesenchymal stem cells from many donors, such as normoglycemic, insulin-resistant, prediabetic, and diabetic ones.

### 3.4. Overall Conclusions and Clinical Output

Our research clearly shows that HG is a strong stimulus for the insulin pathway in visceral Ads. Moreover, it points that GLUT4 expression is strongly correlated not only with HG stimulus but also with ADG progression. All together, obtained data suggest a critical role of GLUT4 in proper Ads formation and glucose homeostasis. What is more, the present findings confirm that each of the analyzed PI3K/AKT pathway molecules has been modulated during ADG and the whole pathway is tightly connected with human visceral ADG. Therefore, it should be pointed out that, analyzed molecules are targets not only of insulin signaling but also of the multiple pathways connected with proliferation, ADG, inflammation, lipid synthesis, and gene expression regulation [8,40]. A particularly interesting area for future research seems to be an investigation of a cross-talk between PI3K/AKT molecules’ expression changes presented in this paper and molecules of other pathways, including meta-inflammation. Due to the fact that meta-inflammation not only initiates abnormal development of Ads mediated at least partially, through WNT/β-catenin signaling, but also leads to the activation of serine kinases, including c-jun N-terminal kinase (JNK), which disrupt downstream insulin signaling [62,63]. Crucial role in meta-inflammation play pro-inflammatory cytokines (like TNF-α, IL-1, IL-6) and inflammasomes (like NLRP3) [4,64].

Furthermore, our results emphasize the importance of maintaining normoglycemia. Even though presented results show in vitro effects in Ads, they may suggest to some extent the importance of avoiding glycemic fluctuations and maintaining healthy body weight. Especially important seems to be the reduction of VAT mass. This stays in line with earlier findings which show that due to the fact that an excess of VAT may not significantly affect the BMI and waist circumference measurement, there is a subgroup of obese patients which are characterized by a normal weight and increased risk of T2DM development, dyslipidemia, and fatty liver disease (metabolically obese, normal weight phenotype of obesity) [65].

Lastly, our study may provide some novel information associated with metabolic memory, an epigenetics-based mechanism of maintaining the effect of HG. Recent data suggest that miRs are involved in Ads development and obesity-associated insulin resistance. Our results also indicate a strong influence of miR molecules on insulin signaling, thus suggesting some new promising potential biomarkers. However, further understanding of how HG affects insulin action using these molecules is crucial for developing future pharmaceutical and nutritional strategies to struggle with metabolic disorders like T2DM.

## 4. Materials and Methods

### 4.1. Cell Culture

Human visceral pAds (HPA-v, ScienCell Research Laboratories, Carlsbad, CA, USA) were cultured as described in [7]. pAds’ donor was a healthy, 45-years old Caucasian woman. Cells from passages 2–5 were used.In brief, the cell culture included 3 subsequent stages: (a) proliferation (5 days), (b) differentiation (12 days) and (c) maturation (6 days). To examine each of the three stages, a part of the cells at the end of each stage were collected and analyzed, while the rest entered further stages (Table 2). During each culture stage cells were maintained in pAdsMedium—PAM (during proliferation stage), pAdsDifferentiation Medium—PADM (during differentiation stage), and AdsMedium—AdM (during maturation stage). All reagents for cell culture were obtained from ScienCell Research Laboratories, Carlsbad, CA, USA. Three independent experiments of cell culture were conducted.The progress of ADG was monitored by cell staining with BODIPY 505/515 (Life Technologies, Eugene, OR, USA).

To assess the effect of HG at each stage, cells were maintained in NG, CHG, or IHGduring the culture. NG means that cells during all three stages of ADG were maintained in a medium with a 5.5 mM concentration of glucose. CHG means that cells during all stages of ADG were maintained in a medium supplemented with glucose (d-(+)-Glucose, Sigma-Aldrich, Saint Louis, MO, USA) to obtain a final concentration of 30mM. IHG means that cells were shifted between NG and HG between stages of ADG (e.g., NHN—means that proliferationwas performed under NG, differentiation- under HG, maturation- under NG).

Chronic HG reflects a diabetic patient, chronic NG mimics a healthy and normoglycemic subject and IHG variants were used to mimic patients with glycemic fluctuations.

### 4.2. RNA Isolation and mRNA Expression Profiling

RNA from each cells variant was isolated using an AllPrep DNA/RNA/Protein Mini Kit (QIAGEN, Hilden, Germany) according to the kit manual. Isolated RNA was checked for proper purity and concentration with a Synergy HT microplate reader (Biotek, Winooski, VT, USA). Reverse transcription of obtained RNA was performed with a High Capacity cDNA Reverse Transcription Kit (Applied Biosystems, Foster City, CA, USA). qReal-Time PCR was conducted using TaqMan Gene Expression Master Mix and following TaqMan Gene Expression Assays: *PI3KR1* (Hs00933163), *PI3KC* (Hs00927728), *IRS1* (Hs00178563), *AKT2* (Hs01086102), *PTEN* (Hs02621230), *GLUT4* (Hs00168966), (Applied Biosystems, Foster City, CA, USA). The Real-Time PCR thermal cycling conditions were as follows: hold (50 °C, 2 min), hold (95 °C, 10 min), 40 cycles (95 °C, 15 s), hold (60 °C, 1 min). For data normalization, we used the 2^−ΔCt^method and the arithmetic average of Ct values obtained for 2 control genes: RLPLO (ribosomal protein lateral stalk subunit P0) and UBC (ubiquitin C). The selection of reference genes was based on the expression-profiling of 16 commonly used reference genes in each study variant using TaqMan Array Human Endogenous Control Plates (Applied Biosystems, Foster City, CA, USA). Obtained data were analyzed using the RefFinder algorithm [66].

### 4.3. Protein Isolation and Expression Profiling

Total protein was isolated with RIPA buffer with the addition of Pierce mini protease and phosphatase inhibitors tablets (Thermo Scientific, Rockford, IL, USA). Shortly, harvested cells were washed three times with DPBS and then incubated with RIPA buffer to cell lysis and protein extraction. Protein concentration in the obtained supernatant was measured with a Protein Determination Kit (Caymann, Ann Arbor, MI, USA). The assay involves the BCA method. In brief, 10× diluted protein samples were incubated with Working Reagent (30 min, 37 °C) and then absorbance was measured at 562 nm. Equalized amount of total protein (adjusted experimentally to each target protein) was used to perform ELISA assays (IRS1-SEC546Hu, AKT2-SEB719Hu, PTEN-SEF822HU, GLUT4-SEC023Hu Cloud-Clone Corp, Katy, TX, USA; PI3K-SL1388Hu Antibody-Sunlong Biotech Co., Ltd., Hangzhou Zheijiang, China). Assays were performed according to the kits manuals.

### 4.4. miRs Isolation and Expression Profiling

The selection of miRs was based on screening analysis, literature research, and the use of bioinformatics tools.

Total miRsfrom cells were isolated using miRVANA Isolation Kit (Applied Biosystems, Vilnius, Lithuania). Reverse transcription was performed with reagents from Applied Biosystems (Foster City, CA, USA). Expression profiling was performed with TaqMan Low-Density Arrays (TLDA) cards (Applied Biosystems, Foster City, CA, USA) in a 7900HT Fast Real-Time PCR System (Applied Biosystems, Foster City, CA, USA). Data were normalized using the 2^−ΔCt^method with the arithmetic average of Ct values for 2 reference genes: U6 and let-7b-5p. Assay IDs (Applied Biosystems, Foster City, CA, USA) for analyzed molecules were as follows: hsa-let-7b-5p (002619) and U6 (001973), hsa-miR-29a-3p (002112), hsa-miR-143-3p (002249), hsa-miR-145-5p (002278), hsa-miR-152-3p (00475), hsa-miR-186-5p (002285), hsa-miR-370-3p (002275), hsa-miR-374b-5p (00563).

### 4.5. Statistical Analysis

Statistical analysis of expression changes was performed using GraphPad Prism 7.0 program. All data were expressed as mean ± SEM.A two-tailed *t*-test was exploited to test the statistical significance between every two mean values. In multiple pair-wise comparisons (N vs. NN vs. NNN and H vs. HH vs. HHH) we used one-way ANOVA with a post-hoc Tukey test. We regarded *p* ≤ 0.05 as significant.

## Figures and Tables

**Table 1:** Analyzed miRs associated with their target genes of insulin signaling.

Mir	Target Genes	References
29a-3p	*IRS*, *AKT*, *PI3KR*, *PTEN*, *GLUT4*	[18,20,21,22,23,24,25,26,27,28]
143-3p	*AKT*, *GLUT4*	[18]
152-3p	*IRS*, *PTEN*	[29,30]
186-5p	*PI3KR*, *PTEN*	[31,32]
370-3p	*IRS*	[33]
374b-5p	*IRS*, *PI3KC*, *PTEN*	[34,35,36,37]

**Table 2:** Glycemic conditions at each step of cell culture for tested variants of HPA-v.

Proliferation	Differentiation	Maturation
N	-	-
H	-	-
N	N	-
N	H	-
H	N	-
H	H	-
N	N	N
N	N	H
N	H	H
N	H	N
H	H	H
H	H	N
H	N	H
H	N	N

N-normoglycemia, H-hyperglycemia, “-”—cells not cultured at this stage.

## Data Availability

Not applicable.

## Abbreviations

ADG	adipogenesis
Ads	adipocytes
AKT	protein kinase B
AT	adipose tissue
CHG	chronic hyperglycemia
C/EBPs	CCAAT enhancer-binding proteins
FOXO1	forkhead box O1
GLUT4	glucose transporter 4
HG	hyperglycemia
IHG	intermittent hyperglycemia
mTOR2	mammalian target of rapamycin 2
NG	normoglycemia
pAds	preadipocytes
PDK1	phosphoinositide-dependent kinase 1
PIP2	phophatidylinositol-4,5-biphospate
PIP3	phophatidylinositol-3,4,5-triphospate
PI3K	phosphatidylinositol-4,5-biphosphate 3-kinase
PI3K-C	phosphatidylinositol-4,5-biphosphate 3-kinase catalytic subunit
PI3K-R	phosphatidylinositol-4,5-biphosphate 3-kinase regulatory subunit
PPARG	peroxisome proliferator-activated receptor gamma
PTEN	phosphatase and tensin homolog
T2DM	type 2 diabetes mellitus
VAT	visceral adipose tissue

## References

[1] Gastaldelli A., Gaggini M., DeFronzo R.A. (2017). Role of Adipose Tissue Insulin Resistance in the Natural History of Type 2 Diabetes: Results from the San Antonio Metabolism Study. Diabetes.

[2] Grundy S.M. (2015). Adipose tissue and metabolic syndrome: Too much, too little or neither. Eur. J. Clin. Investig..

[3] Wróblewski A., Strycharz J., Świderska E., Balcerczyk A., Szemraj J., Drzewoski J., Śliwińska A. (2021). Chronic and Transient Hyperglycemia Induces Changes in the Expression Patterns of *IL6* and *ADIPOQ* Genes and Their Associated Epigenetic Modifications in Differentiating Human Visceral Adipocytes. Int. J. Mol. Sci..

[4] Deng T., Lyon C.J., Bergin S., Caligiuri M.A., Hsueh W.A. (2016). Obesity, Inflammation, and Cancer. Annu. Rev. Pathol..

[5] Swiderska E., Podolska M., Strycharz J., Szwed M., Abramczyk H., Brozek-Pluska B., Wroblewski A., Szemraj J., Majsterek I., Drzewoski J. (2019). Hyperglycemia Changes Expression of Key Adipogenesis Markers (C/EBPα and PPARγ) and Morphology of Differentiating Human Visceral Adipocytes. Nutrients.

[6] Ishaq A., Dufour D., Cameron K., von Zglinicki T., Saretzki G. (2018). Metabolic memory of dietary restriction ameliorates DNA damage and adipocyte size in mouse visceral adipose tissue. Exp. Gerontol..

[7] Strycharz J., Swiderska E., Wroblewski A., Podolska M., Czarny P., Szemraj J., Balcerczyk A., Drzewoski J., Kasznicki J., Sliwinska A. (2018). Hyperglycemia Affects miRNAs Expression Pattern during Adipogenesis of Human Visceral Adipocytes-Is Memorization Involved?. Nutrients.

[8] Vanhaesebroeck B., Stephens L., Hawkins P. (2012). PI3K signalling: The path to discovery and understanding. Nat. Rev. Mol. Cell Biol..

[9] Świderska E., Strycharz J., Wróblewski A., Szemraj J., Drzewoski J., Śliwińska A. (2018). Role of PI3K/AKT pathway in insulin-mediated glucose uptake. Blood Glucose Levels.

[10] Li A., Qiu M., Zhou H., Wang T., Guo W. (2017). PTEN, Insulin Resistance and Cancer. Curr. Pharm. Des..

[11] Gao Y., Moten A., Lin H.-K. (2014). Akt: A new activation mechanism. Cell Res..

[12] Fischer-Posovszky P., Tews D., Horenburg S., Debatin K.M., Wabitsch M. (2012). Differential function of Akt1 and Akt2 in human adipocytes. Mol. Cell Endocrinol..

[13] Baudry A., Yang Z.Z., Hemmings B.A. (2006). PKBalpha is required for adipose differentiation of mouse embryonic fibroblasts. J. Cell Sci..

[14] Cai H., Dong L.Q., Liu F. (2016). Recent Advances in Adipose mTOR Signaling and Function: Therapeutic Prospects. Trends Pharmacol. Sci..

[15] Nakae J., Kitamura T., Kitamura Y., Biggs W.H., Arden K.C., Accili D. (2003). The Forkhead Transcription Factor Foxo1 Regulates Adipocyte Differentiation. Dev. Cell.

[16] Andersen E., Ingerslev L.R., Fabre O., Donkin I., Altintas A., Versteyhe S., Bisgaard T., Kristiansen V.B., Simar D., Barres R. (2019). Preadipocytes from obese humans with type 2 diabetes are epigenetically reprogrammed at genes controlling adipose tissue function. Int. J. Obes..

[17] Ramzan F., D’Souza R.F., Durainayagam B.R., Milan A.M., Markworth J.F., Miranda-Soberanis V., Sequeira I.R., Roy N.C., Poppitt S.D., Mitchell C.J. (2020). Circulatory miRNA biomarkers of metabolic syndrome. Acta Diabetol..

[18] Herrera B.M., Lockstone H.E., Taylor J.M., Ria M., Barrett A., Collins S., Kaisaki P., Argoud K., Fernandez C., Travers M.E. (2010). Global microRNA expression profiles in insulin target tissues in a spontaneous rat model of type 2 diabetes. Diabetologia.

[19] Landrier J.F., Derghal A., Mounien L. (2019). MicroRNAs in Obesity and Related Metabolic Disorders. Cells.

[20] Yang W.M., Jeong H.J., Park S.Y., Lee W. (2014). Induction of miR-29a by saturated fatty acids impairs insulin signaling and glucose uptake through translational repression of IRS-1 in myocytes. FEBS Lett..

[21] Liu T., Sun Y.C., Cheng P., Shao H.G. (2019). Adipose tissue macrophage-derived exosomal miR-29a regulates obesity-associated insulin resistance. Biochem. Biophys. Res. Commun..

[22] Esteves J.V., Enguita F.J., Machado U.F. (2017). MicroRNAs-Mediated Regulation of Skeletal Muscle GLUT4 Expression and Translocation in Insulin Resistance. J. Diabetes Res..

[23] Chen L., Song J., Cui J., Hou J., Zheng X., Li C., Liu L. (2013). microRNAs regulate adipocyte differentiation. Cell Biol. Int..

[24] He A., Zhu L., Gupta N., Chang Y., Fang F. (2007). Overexpression of micro ribonucleic acid 29, highly up-regulated in diabetic rats, leads to insulin resistance in 3T3-L1 adipocytes. Mol. Endocrinol..

[25] Boudreau R.L., Jiang P., Gilmore B.L., Spengler R.M., Tirabassi R., Nelson J.A., Ross C.A., Xing Y., Davidson B.L. (2014). Transcriptome-wide discovery of microRNA binding sites in human brain. Neuron.

[26] Grosswendt S., Filipchyk A., Manzano M., Klironomos F., Schilling M., Herzog M., Gottwein E., Rajewsky N. (2014). Unambiguous identification of miRNA:target site interactions by different types of ligation reactions. Mol. Cell.

[27] Kameswaran V., Bramswig N.C., McKenna L.B., Penn M., Schug J., Hand N.J., Chen Y., Choi I., Vourekas A., Won K.J. (2014). Epigenetic regulation of the DLK1-MEG3 microRNA cluster in human type 2 diabetic islets. Cell Metab..

[28] Kong G., Zhang J., Zhang S., Shan C., Ye L., Zhang X. (2011). Upregulated microRNA-29a by hepatitis B virus X protein enhances hepatoma cell migration by targeting *PTEN* in cell culture model. PLoS ONE.

[29] Wang S., Wang L., Dou L., Guo J., Fang W., Li M., Meng X., Man Y., Shen T., Huang X. (2016). MicroRNA 152 regulates hepatic glycogenesis by targeting *PTEN*. FEBS J..

[30] Xu Q., Jiang Y., Yin Y., Li Q., He J., Jing Y., Qi Y.-T., Xu Q., Li W., Lu B. (2013). A regulatory circuit of miR-148a/152 and *DNMT1* in modulating cell transformation and tumor angiogenesis through IGF-IR and IRS1. J. Mol. Cell Biol..

[31] Xiang Y., Chen Y.-J., Yan Y.-B., Liu Y., Qiu J., Tan R.-Q., Tian Q., Guan L., Niu S.-S., Xin H.-W. (2020). MiR-186 bidirectionally regulates cisplatin sensitivity of ovarian cancer cells via suppressing targets *PIK3R3* and *PTEN* and upregulating *APAF1* expression. J. Cancer.

[32] Feng H., Zhang Z., Qing X., French S.W., Liu D. (2019). miR-186-5p promotes cell growth, migration and invasion of lung adenocarcinoma by targeting *PTEN*. Exp. Mol. Pathol..

[33] Hou W.Z., Chen X.L., Wu W., Hang C.H. (2017). MicroRNA-370-3p inhibits human vascular smooth muscle cell proliferation via targeting KDR/AKT signaling pathway in cerebral aneurysm. Eur. Rev. Med. Pharmacol. Sci..

[34] Xue Y., Ouyang K., Huang J., Zhou Y., Ouyang H., Li H., Wang G., Wu Q., Wei C., Bi Y. (2013). Direct conversion of fibroblasts to neurons by reprogramming PTB-regulated microRNA circuits. Cell.

[35] Skalsky R.L., Corcoran D.L., Gottwein E., Frank C.L., Kang D., Hafner M., Nusbaum J.D., Feederle R., Delecluse H.J., Luftig M.A. (2012). The viral and cellular microRNA targetome in lymphoblastoid cell lines. PLoS Pathog..

[36] Kishore S., Jaskiewicz L., Burger L., Hausser J., Khorshid M., Zavolan M. (2011). A quantitative analysis of CLIP methods for identifying binding sites of RNA-binding proteins. Nat. Methods.

[37] Damania B., Haecker I., Gay L.A., Yang Y., Hu J., Morse A.M., McIntyre L.M., Renne R. (2012). Ago HITS-CLIP Expands Understanding of Kaposi’s Sarcoma-associated Herpesvirus miRNA Function in Primary Effusion Lymphomas. PLoS Pathog..

[38] Leonardini A., Laviola L., Perrini S., Natalicchio A., Giorgino F. (2009). Cross-Talk between PPARgamma and Insulin Signaling and Modulation of Insulin Sensitivity. PPAR Res..

[39] Guo S. (2014). Insulin signaling, resistance, and the metabolic syndrome: Insights from mouse models into disease mechanisms. J. Endocrinol..

[40] Huang X., Liu G., Guo J., Su Z. (2018). The PI3K/AKT pathway in obesity and type 2 diabetes. Int. J. Biol. Sci..

[41] Varma S., Lal B.K., Zheng R., Breslin J.W., Saito S., Pappas P.J., Hobson R.W., Duran W.N. (2005). Hyperglycemia alters PI3k and Akt signaling and leads to endothelial cell proliferative dysfunction. Am. J. Physiol. Heart Circ. Physiol..

[42] Hernandez R., Teruel T., Lorenzo M. (2001). Akt mediates insulin induction of glucose uptake and up-regulation of *GLUT4* gene expression in brown adipocytes. FEBS Lett..

[43] Tremblay F., Lavigne C., Jacques H., Marette A. (2001). Defective Insulin-Induced GLUT4 Translocation in Skeletal Muscle of High Fat–Fed Rats Is Associated with Alterations in both Akt/Protein Kinase B and Atypical Protein Kinase C (ζ/λ) Activities. Diabetes.

[44] Xi G., Shen X., Wai C., White M.F., Clemmons D.R. (2019). Hyperglycemia induces vascular smooth muscle cell dedifferentiation by suppressing insulin receptor substrate-1-mediated p53/KLF4 complex stabilization. J. Biol. Chem..

[45] Wang Y., He G., Wang F., Zhang C., Ge Z., Zheng X., Deng H., Yuan C., Zhou B., Tao X. (2019). Aspirin inhibits adipogenesis of tendon stem cells and lipids accumulation in rat injury tendon through regulating PTEN/PI3K/AKT signalling. J. Cell Mol. Med..

[46] Meugnier E., Faraj M., Rome S., Beauregard G., Michaut A., Pelloux V., Chiasson J.L., Laville M., Clement K., Vidal H. (2007). Acute hyperglycemia induces a global downregulation of gene expression in adipose tissue and skeletal muscle of healthy subjects. Diabetes.

[47] Xi G., Wai C., White M.F., Clemmons D.R. (2017). Down-regulation of Insulin Receptor Substrate 1 during Hyperglycemia Induces Vascular Smooth Muscle Cell Dedifferentiation. J. Biol. Chem..

[48] Nelson B.A., Robinson K.A., Buse M.G. (2000). High glucose and glucosamine induce insulin resistance via different mechanisms in 3T3-L1 adipocytes. Diabetes.

[49] Buren J., Liu H., Lauritz J., Eriksson J. (2003). High glucose and insulin in combination cause insulin receptor substrate-1 and -2 depletion and protein kinase B desensitisation in primary cultured rat adipocytes: Possible implications for insulin resistance in type 2 diabetes. Eur. J. Endocrinol..

[50] Back K., Islam R., Johansson G.S., Chisalita S.I., Arnqvist H.J. (2012). Insulin and IGF1 receptors in human cardiac microvascular endothelial cells: Metabolic, mitogenic and anti-inflammatory effects. J. Endocrinol..

[51] Laybutt D.R., Chisholm D.J., Kraegen E.W. (1997). Specific adaptations in muscle and adipose tissue in response to chronic systemic glucose oversupply in rats. Am. J. Physiol. Endocrinol. Metab..

[52] Chouchani E.T., Kajimura S. (2019). Metabolic adaptation and maladaptation in adipose tissue. Nat. Metab..

[53] Chuang C.C., Yang R.S., Tsai K.S., Ho F.M., Liu S.H. (2007). Hyperglycemia enhances adipogenic induction of lipid accumulation: Involvement of extracellular signal-regulated protein kinase 1/2, phosphoinositide 3-kinase/Akt, and peroxisome proliferator-activated receptor gamma signaling. Endocrinology.

[54] Masi L.N., Lotufo P.A., Ferreira F.M., Rodrigues A.C., Serdan T.D.A., Souza-Siqueira T., Braga A.A., Saldarriaga M.E.G., Alba-Loureiro T.C., Borges F.T. (2021). Profiling plasma-extracellular vesicle proteins and microRNAs in diabetes onset in middle-aged male participants in the ELSA-Brasil study. Physiol. Rep..

[55] Nusca A., Tuccinardi D., Albano M., Cavallaro C., Ricottini E., Manfrini S., Pozzilli P., Di Sciascio G. (2018). Glycemic variability in the development of cardiovascular complications in diabetes. Diabetes Metab. Res. Rev..

[56] Zhang Z.-Y., Miao L.-F., Qian L.-L., Wang N., Qi M.-M., Zhang Y.-M., Dang S.-P., Wu Y., Wang R.-X. (2019). Molecular Mechanisms of Glucose Fluctuations on Diabetic Complications. Front. Endocrinol..

[57] Shi X.L., Ren Y.Z., Wu J. (2011). Intermittent high glucose enhances apoptosis in INS-1 cells. Exp. Diabetes Res..

[58] Ceriello A., Kilpatrick E.S. (2013). Glycemic variability: Both sides of the story. Diabetes Care.

[59] Testa R., Bonfigli A.R., Prattichizzo F., La Sala L., De Nigris V., Ceriello A. (2017). The “Metabolic Memory” Theory and the Early Treatment of Hyperglycemia in Prevention of Diabetic Complications. Nutrients.

[60] Strycharz J., Wroblewski A., Zieleniak A., Swiderska E., Matyjas T., Rucinska M., Pomorski L., Czarny P., Szemraj J., Drzewoski J. (2021). Visceral Adipose Tissue of Prediabetic and Diabetic Females Shares a Set of Similarly Upregulated microRNAs Functionally Annotated to Inflammation, Oxidative Stress and Insulin Signaling. Antioxidants.

[61] Ying C., Liu T., Ling H., Cheng M., Zhou X., Wang S., Mao Y., Chen L., Zhang R., Li W. (2017). Glucose variability aggravates cardiac fibrosis by altering AKT signalling path. Diabetes Vasc. Dis. Res..

[62] Manning B.D., Cantley L.C. (2007). AKT/PKB signaling: Navigating downstream. Cell.

[63] Aamir K., Khan H.U., Sethi G., Hossain M.A., Arya A. (2020). Wnt signaling mediates TLR pathway and promote unrestrained adipogenesis and metaflammation: Therapeutic targets for obesity and type 2 diabetes. Pharmacol. Res..

[64] Wu K.K., Cheung S.W., Cheng K.K. (2020). NLRP3 Inflammasome Activation in Adipose Tissues and Its Implications on Metabolic Diseases. Int. J. Mol. Sci..

[65] Vecchie A., Dallegri F., Carbone F., Bonaventura A., Liberale L., Portincasa P., Fruhbeck G., Montecucco F. (2018). Obesity phenotypes and their paradoxical association with cardiovascular diseases. Eur. J. Intern. Med..

[66] Xie F., Xiao P., Chen D., Xu L., Zhang B. (2012). miRDeepFinder: A miRNA analysis tool for deep sequencing of plant small RNAs. Plant Mol. Biol..

